# Hyperion Image Analysis Depicts a Preliminary Landscape of Tumor Immune Microenvironment in OSCC with Lymph Node Metastasis

**DOI:** 10.1155/2021/9975423

**Published:** 2021-06-21

**Authors:** Shang Xie, Xin-Yuan Zhang, Xiao-Feng Shan, Vicky Yau, Jian-Yun Zhang, Wei Wang, Yong-Pan Yan, Zhi-Gang Cai

**Affiliations:** ^1^Department of Oral and Maxillofacial Surgery, Peking University School and Hospital of Stomatology & National Clinical Research Center for Oral Diseases & National Engineering Laboratory for Digital and Material Technology of Stomatology & Beijing Key Laboratory of Digital Stomatology, Beijing 100081, China; ^2^Stony Brook University School of Dental Medicine, South Drive, Stony Brook, NY 11794, USA; ^3^Department of Oral Pathology, Peking University School and Hospital of Stomatology, Beijing 100081, China; ^4^Gencode Diagnostics Inc., #7 Liye Road, 5th Floor, Changping District, Beijing 102206, China

## Abstract

**Background:**

Oral squamous cell carcinoma (OSCC) constitutes the most common types of oral cancer. Because its prognosis varies significantly, identification of a tumor immune microenvironment could be a critical tool for treatment planning and predicting a more accurate prognosis. This study is aimed at utilizing the Hyperion imaging system to depict a preliminary landscape of the tumor immune microenvironment in OSCC with lymph node metastasis.

**Methods:**

We collected neoplasm samples from OSCC patients. Their formalin-fixed, paraffin-embedded (FFPE) tissue sections were obtained and stained utilizing a panel of 26 clinically relevant metal-conjugated antibodies. Detection and analysis were performed for these stained cells with the Hyperion imaging system.

**Results:**

Four patients met our inclusion criteria. We depicted a preliminary landscape of their tumor immune microenvironment and identified 25 distinct immune cell subsets from these OSCC patients based on phenotypic similarity. All these patients had decreased expression of CD8^+^ T cells in tumor specimens. Variety in cell subsets was seen, and more immune activated cells were found in patient A and patient B than those in patient C and patient D. Such differences in tumor immune microenvironments can contribute to forecasting of individual prognoses.

**Conclusion:**

The Hyperion imaging system helped to delineate a preliminary and multidimensional landscape of the tumor immune microenvironment in OSCC with lymph node metastasis and provided insights into the influence of the immune microenvironment in determination of prognoses. These results reveal possible contributory factors behind different prognoses of OSCC patients with lymph node metastasis and provide reference for individual treatment planning.

## 1. Introduction

Oral squamous cell carcinoma (OSCC) contributes the most to neoplasms in the oral and maxillofacial region. Its high mortality leads to a heavy burden to the global health system [1]. Despite rapid advancement in treatments, assessment of accurate prognoses for OSCC has entered a bottleneck period and the five-year overall rate has not significantly ameliorated in the past two decades [2, 3]. One potential reason could be the inability of current treatments to change the existing tumor immune microenvironment; the TNM staging system, on which treatment planning is mostly based, remains inaccurate. In clinical practice, it is indeed common for a small part of OSCC patients with lymph node metastasis to have good prognoses. One possible reason might be that the intrinsic heterogeneity of the tumor immune microenvironment has been overlooked by the TNM system for the mismatch between TMN stage classification and clinical advancement. Thus, there is an important clinical need to identify tools for accurate prognosis evaluation and new treatment strategy formulation, especially for patients with lymph node metastasis.

The tumor microenvironment (TME) is comprised of the tumor cells, surrounded by immune cells, fibroblasts, extracellular matrix, signaling molecules, etc. [4, 5]. Tumor cells and the components of TME, including CD8^+^ T cells [6, 7], CD4^+^ T cells [8, 9], NK cells [10], dendritic cells [11, 12], B cells [13, 14], macrophages [15], FOXP3 [16], and Collagen I [17] have close correlations to tumor treatment and prognosis. In past decades, due to the limitations of tools and clinical methodologies, it was difficult to analyze multiple components and assess tumor heterogeneity of the TME. Yet, with the newly developed Hyperion imaging system, we are now able to include such components to determine more accurate prognoses [18].

In this study, we depict a preliminary and multidimensional landscape of the tumor immune microenvironment of OSCC patients with lymph node metastasis and provide insights into its influence in prognosis by utilizing the Hyperion imaging system.

## 2. Materials and Methods

### 2.1. Sample Selection

Medical records of patients admitted for operation to the Peking University Hospital of Stomatology between October, 2018 and April, 2019 were reviewed. In order to focus on specific stages of OSCC (T1-T2, lymph node metastasis) and decrease the clinical heterogeneity, the following criteria were established: (1) 25 to 45 years old, (2) Asian, (3) tumor size less than 2 cm, (4) tumors were located at the tongue, (5) pathological diagnosis were SCC, and (6) tumors have well-moderately differentiated SCC and with lymph node metastasis. No patient has undergone radiotherapy, chemotherapy, or immunotherapy before operation. Tumor tissues were obtained from the Department of Pathology, Peking University Hospital of Stomatology. The included patients were followed up on April 19th, 2021.

### 2.2. Panel and Antibody Conjugation

The antibody panel was designed with 26 protein markers, as described in our previous publication in which descriptions of these antibodies, metal labels, clones, catalog, and vendor can be found [19]. The panel information can be also found in Supplementary S1. Conjugation of metal-conjugated antibodies was performed utilizing a Maxpar labeling kit (Fluidigm, USA).

### 2.3. Tissue Antibody Labeling

Tissue samples were obtained and fixed in formalin and embedded in paraffin. Protocols of tissue antibody labeling were similar to those described in the published articles [19, 20].

### 2.4. Hyperion Tissue Imager Scan

The largest square area of an imager scan is 500 *μ*m × 500 *μ*m. Images were acquired and analyzed utilizing a Hyperion imaging system (Fluidigm) [19, 21, 22].

### 2.5. Data Procedure and Normalization

Data were divided into single cells using CellProfiler v3.1.8 [23]. Detailed data procedures were similar to those in the published article [19]. For t-SNE and PhenoGraph, the data were normalized by Harmony [24].

### 2.6. Clustering and Barnes–Hut t-SNE

Single cells were clustered into groups based on their phenotypical similarity and molecular markers utilizing PhenoGraph [25]. For visualization, high-dimensional data were decreased to two dimensions utilizing the algorithm t-SNE [26–28]. We used the Barnes–Hut achievement of t-SNE to Harmony-normalized data with default parameters (perplexity = 42).

## 3. Results

### 3.1. Characteristics of OSCC Patients

After reviewing, four OSCC patients met our inclusion criteria. Their clinical characteristics, treatment strategies, and follow-up information are shown in Table 1. All four patients underwent extensive resection of tongue neoplasm and neck lymph node dissection. During follow-up, one patient (patient C, female) died of tumor recurrence at 21 months after the operation and this patient had no history of smoking and drinking.

### 3.2. Sample Procedure and Data Acquisition

**Figure 1:**
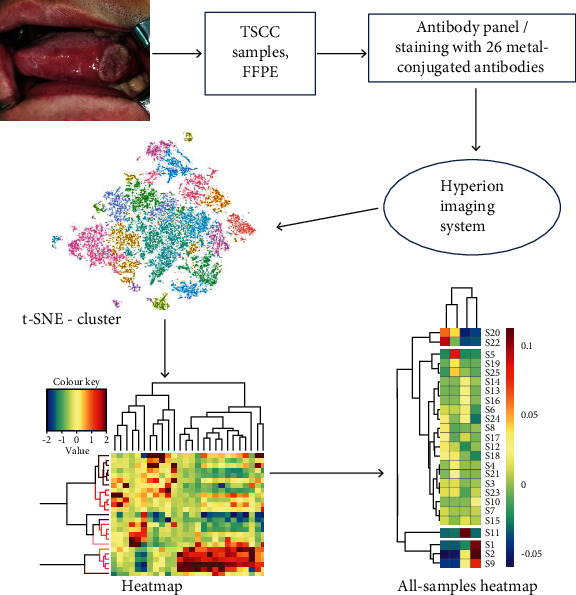
Overall study design. Hyperion imaging system was utilized to detect OSCC tissue samples, and the output data were used for cluster analyses. TSCC: tongue squamous cell carcinoma; FFPE: formalin-fixed, paraffin-embedded.

All sample slices were marked by a panel of 26 metal-labeled antibodies. Labeling antibodies were detected, and the data were analyzed by the Hyperion imaging system. The scheme of the overall study design is shown in Figure 1.

### 3.3. Analysis of Immune Phenotypes of OSCC Samples by Hyperion Imaging System

**Figure 2:**
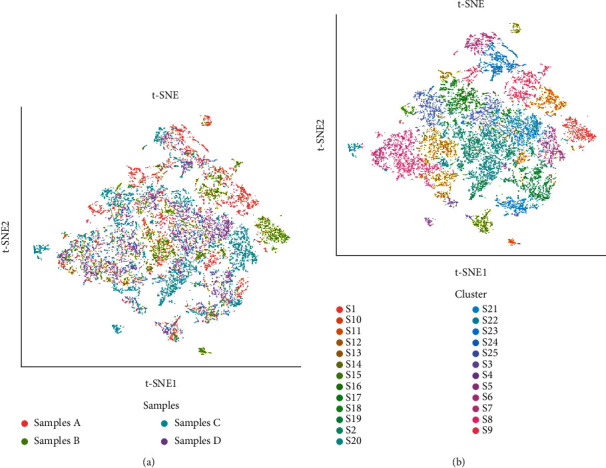
Cluster analysis of OSCC samples. (a) Single cells from each PhenoGraph cluster in four samples of OSCC with lymph node metastasis displayed by t-SNE descending dimension map were colored-coded in heat map images. The colors represent different samples. (b) t-SNE descending dimension map displays single cells from each PhenoGraph cluster. They are identified in heat map images colored according to individual cluster. Colors represent different clusters of immune cells. OSCC: oral squamous cell carcinoma.

These metal antibody-labeled slices were scanned to identify different markers of tumor cells, lymphocyte types, vasculature, and structures of tissue cells by the Hyperion imaging system. To visualize the characteristics of TME among the four OSCC patients (patients A, B, C, and D), a t-SNE map was firstly utilized to illustrate single cells from each PhenoGraph cluster according to samples (Figure 2(a)). This panel showed that the components in TME were obviously distinctive among these patients despite similar clinicopathological characteristics, indicating differences in their future prognoses.

To visualize the panorama of immune cell subsets and extracellular matrix among OSCC patients with lymph node metastasis, we also showed a t-SNE map according to different clusters of cell subsets. In this panorama, we identified 25 unequal cell clusters in TME based on phenotypic similarity (Figure 2(b)).

**Figure 3:**
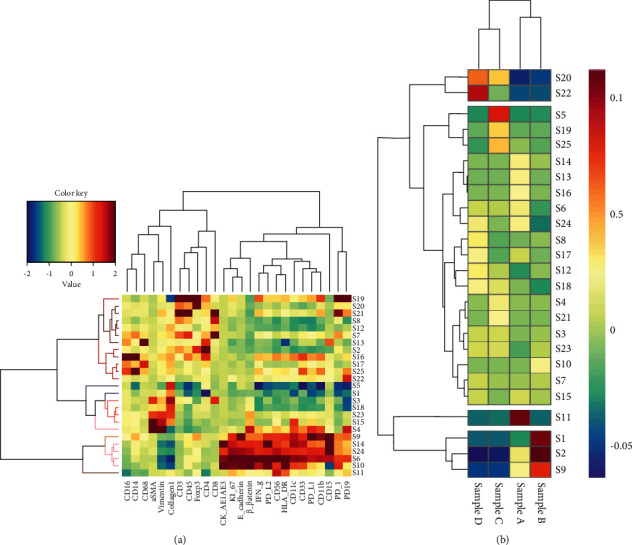
Heat map of OSCC samples. (a) The heat map shows the differential expression of the panel markers of 25 subsets. Clusters and markers are grouped according to expression profiles. Certain cell clusters are identified as known immune cell types according to typically expressed markers. (b) Differential cluster analyses of the 4 OSCC samples with lymph node metastasis. OSCC: oral squamous cell carcinoma.

To further explore these cell clusters, we further visualized these markers via heat map. Clusters and tumor markers were grouped based on expression profiles, and the result is shown in Figure 3(a). From the heat map, we inferred 25 different cell clusters representing the CD8^+^ T cell subsets, CD4^+^ T cell subsets, Collagen I, CD19^+^ B cell subsets, CD56^+^ NK cell subsets, and FOXP3 cell subsets.

Based on differences in cell clusters, cluster and patient analyses were performed (Figure 3(b)). From Figure 3(b), we found that cell clusters had a significant disparity among different patients, but all CD8^+^ cell populations were found to be underexpressed in all included patients, implying that all of them had an incomplete tumor immune microenvironment.

Notably, CD56^+^ NK cells were highly expressed in patient A and CD4^+^ in patient B; FOXP3 and CD19 were close to completely absent in both. Collagen I was highly expressed with less expression of CD4^+^ cells in patient C, and FOXP3 and CD19 were highly expressed while CD4^+^ cells were absent in patient D. It suggested the existence of more immunosuppressive cells and less immune activation cells in patient C and patient D.

Taken together, it was hinted that patient A and patient B might have better prognoses than patient C and patient D, reflecting that a thorough classification of OSCC patients might be beneficial for accurate prognostic assessment of OSCC and implementation of precisely targeted therapies.

## 4. Discussion

Previous studies demonstrated that tumor cells, immune cells, signaling molecules, and extracellular matrix have mutual interactions in TME [29, 30]. Therefore, understanding differences of tumor immune microenvironment components among patients would provide pivotal information for evaluating prognosis and identifying individualized treatments. This study is first of a new focus to depict a landscape of TME in OSCC patients with lymph node metastasis using the Hyperion imaging system.

The Hyperion imaging system is a new scan and analytical tool for analyses of heterogeneity in TME, and it allows simultaneous detection of dozens of metal-labeled antibodies in the same tissue slices. Currently, it has been successfully used to show the components of many tumor microenvironments [22, 31, 32] and explore the immune response of COVID-19 patients [20]. However, to date, there has been no publication on the use of the Hyperion imaging system to scan and analyze the heterogeneity of OSCC with lymph node metastasis. Therefore, our study is aimed at filling in the gap by exploring and analyzing the intratumoral heterogeneity of OSCC with lymph node metastasis, and thereby suggesting strategies for prognosis and precision treatment planning.

In this study, we depicted a landscape of TME according to a panel of 26 antibodies. In this landscape, we identified several critical immune cell phenotypes including CD8^+^ T cells, CD4^+^ T cells, CD56^+^ NK cells, CD19^+^ B cells, FOXP3^+^ cells, and Collagen I cell subsets.

Previous publications had demonstrated that cytotoxic CD8^+^ T cells targeted and killed tumor cells [6, 7, 33]. In our study, we found low expression of CD8^+^ T cells in all included patients, suggesting their poorly activated immune systems. The prediction of poor prognoses in general for these patients is consistent with the publication of Fang et al. [34].

As we all know, CD4^+^ T cells are important for the activation and expansion of CD8^+^ effectors, and also vital for the maintenance and generation of CD8^+^ T cell memory [8]. In the TME, they might also participate to amplify the CD8^+^ T cell response and have the ability to control tumor growth in different ways [8]. In our study, we found that CD4^+^ T cells were highly expressed in patient B but poorly expressed in patient C and patient D, implying that patient C and patient D had lower immunity.

Natural killer cells (NK cells) are also important immune cells, and many authors proposed that the presence of NK cells was correlated to favorable prognosis of OSCC. For example, Ikeda et al. reported that even a small quantity of NK cells were still helpful to determine prognosis of advanced OSCC [35]. In our study, high expression of CD56 NK cells in patient A compared to that in patient C and patient D suggested a better relative prognosis in the patient.

Classically, B cells are deemed to positively regulate immune responses and boost T cell activation and proliferation via antigen presentation [36]. However, numerous studies recently reported that B cells were relevant to unfavorable prognosis and advancement of lymph node metastasis [14, 37]. In this study, B cells were more highly expressed in patient D but poorly expressed in both patient A and patient B. Besides, the presence of high numbers of FOXP3^+^ T cells in cancer tissues predicts worse relapse-free survival and decreased overall survival [16]. Also highly expressed in patient D, FOXP3^+^ might contribute to worse prognosis in this patient comparing to those of patient A and patient B.

Previous publications have reported that Collagen I increased tumorigenesis, promoted cancer metastasis by upregulating N-cadherin expression and increasing motility [38], and promoted epithelial-to-mesenchymal transition by TGF-*β* signaling [39]. In our study, Collagen I was highly expressed in patient C but no obvious expressions were seen in other patients, suggesting that patient C had the worse immunosuppressive TME. Notably, patient C died of tumor metastasis at 21 months after the surgical operation, potentially caused by her poor tumor microenvironment.

Based on these data, we could deduce that although all patients demonstrated similar clinical characteristics, patient A and B might have potentially better prognoses due to the presence of more immune activated cells and less immunosuppressive cells in their tumor microenvironment.

The current study is a very early study to analyze heterogeneity and depict the landscape of the tumor immune microenvironment of OSCC using the Hyperion imaging system. Hence, further studies would definitely be required to address the following limitations. Firstly, since current data were based on limited numbers of patients, the expression tendency for the abovementioned markers and cell subsets would need further investigation to look for consistency. Secondly, the phenotypes and immune cell clusters were displayed according to bioinformatics analysis and have not been identified by cell and animal experiments. Besides, the fee for utilizing the Hyperion imaging system could be very challenging to investigate individual cases. Based on these limitations, further studies are required to increase the sample size and identify the roles of these phenotypes with a low-cost approach in the future.

## 5. Conclusion

The Hyperion imaging system depicted a preliminary and multidimensional immune microenvironment landscape for OSCC with lymph node metastasis and provided insights into the influence of the heterogeneity and variable immune cell subsets on determination of prognoses. These results reveal possible factors which contribute to different prognoses in spite of the similar lymph node involvement and clinical characteristics. Furthermore, long term follow-up will be needed in the future, and further study for targeted therapy will greatly improve individualization of OSCC treatments.

## Figures and Tables

**Table 1:** Clinicopathological characteristics of the 4 included patients.

Study NO	Demographic information	Tumor site	Tumor size	TNM stage	Smoking	Alcohol	Pathological features	Treatment strategy	Follow-up (months)
Patient A	Male, 36 years, Asian	Tongue	Ulceration, diameter 2.0 cm	T2N1M0	Yes	Yes	Well-moderately differentiated SCC, lymph node metastasis, 1.0 cm depth of invasion, invasion of striated muscles	Operation: extensive resection of tongue cancer and cervical lymph node dissection; radiotherapy but without chemotherapy	No recurrence, 25 months
Patient B	Male, 39 years, Asian	Tongue	Ulceration, diameter 1.3 cm	T1N1M0	Yes	No	Well differentiated SCC, lymph node metastasis, 0.2 cm depth of invasion	Operation: extensive resection of tongue cancer and cervical lymph node dissection; without radiotherapy and chemotherapy	No recurrence, 24 months
Patient C	Female, 41 years, Asian	Tongue	Ulceration, diameter 1.3 cm	T2N1M0	No	No	Well differentiated SCC, lymph node metastasis, 0.6 cm depth of invasion, invasion of striated muscles and nerves	Operation: extensive resection of tongue cancer and cervical lymph node dissection; without radiotherapy and chemotherapy	Death, 21 months
Patient D	Female, 29 years, Asian	Tongue	Ulceration, diameter 1.8 cm	T2N1M0	No	No	Well differentiated SCC, lymph node metastasis, 1.0 cm depth of invasion	Operation: extensive resection of tongue cancer and cervical lymph node dissection; without radiotherapy and chemotherapy	No recurrence, 26 months

TNM stage: T = tumor; N = node; M = metastasis. SCC: squamous cell carcinoma.

## Data Availability

Data are available from corresponding author Zhi-Gang Cai (c2013xs@163.com) or first author Shang Xie (xs2013@hsc.pku.edu.cn).

## Supplementary Materials

Supplementary Materials S1: antibodies targeting tumor immune environment of OSCC.
