# Supplementary material for: Hyperion Image Analysis Depicts a Preliminary Landscape of Tumor Immune Microenvironment in OSCC with Lymph Node Metastasis
Source: J Immunol Res. 2021 Jun 21;2021:9975423. doi: 10.1155/2021/9975423 (PMC8238606; doi:10.1155/2021/9975423)
Supplement: Supplementary Materials — S1: antibodies targeting tumor immune environment of OSCC. [file 9975423.f1.docx]

| Supplementary S1 **Antibodies targeting tumor immune environment of OSCC** | | | | |
| --- | --- | --- | --- | --- |
| **Antibody** | **Clone** | **Vendor** | **Catalog** | **Label** |
| CD45 | 2D1 | Biolegend | 368502 | Er166 |
| CD3 | Polyclonal, C-Termina | Fluidigm | 3170019D | Er170 |
| CD4 | EPR6855 | Fluidigm | 3156033D | Gd156 |
| Foxp3 | 206D | Biolegend | 320102 | Gd155 |
| CD8a | D8A8Y | Fluidigm | 3162035D | Dy162 |
| CD11b/Mac-1 | EPR1344 | Fluidigm | 3149028D | Sm149 |
| CD11c | Polyclonal | Fluidigm | 3154025D | Sm154 |
| CD14 | EPR3653 | Fluidigm | 3144025D | Nd144 |
| CD15 | HI98 | Biolegend | 301902 | Eu153 |
| CD16 | EPR16784 | Fluidigm | 3146020D | Nd146 |
| CD19 | 6OMP31 | Fluidigm | 3142014D | Nd142 |
| CD33 | Polyclonal | Fluidigm | 3145017D | Nd145 |
| CD56 | RNL-1 | Abcam | 9018 | Sm152 |
| CD68 | KP1 | Biolegend | 916104 | Tb159 |
| IFN-γ | Polyclonal | Abcam | 9657 | Sm147 |
| HLA-DR | YE2/36 HLK | Fluidigm | 3174023D | Yb174 |
| CD274/PD-L1 | E1L3N | Fluidigm | 3150031D | Nd150 |
| CD273/PD-L2 | 176611 | Fluidigm | 3172028D | Yb172 |
| CD279/PD-1 | EPR4877(2) | Fluidigm | 3165039D | Ho165 |
| CK AE1/AE3 | AE-1/AE-3 | Biolegend | 914204 | Dy164 |
| α-SMA | 1A4 | ebioscience | 14-9760-82 | Pr141 |
| Collagen I | 3G3 | Abcam | 88147 | Tm169 |
| CD324/E-Cadherin | 4A2 | Abcam | 231303 | Gd158 |
| Vimentin | RV202 | Abcam | 8978 | Nd148 |
| Ki-67 | Ki-67 | Biolegend | 350502 | Er168 |
| β-catenin | 12F7 | Biolegend | 844603 | Lu175 |
